# The complete chloroplast genome sequence and phylogenetic analysis of Ilex × Koehneana ‘Wirt L. Winn’ (Aquifoliaceae)

**DOI:** 10.1080/23802359.2021.1882904

**Published:** 2021-03-11

**Authors:** Xinran Chong, Yunlong Li, Yanwei Zhou, Hong Chen, Xinzhi Li, Naiwei Li, Xiaoqing Lu, Fan Zhang

**Affiliations:** a Jiangsu Key Laboratory for the Research and Utilization of Plant Resources, Institute of Botany, Jiangsu Province and Chinese Academy of Sciences, Nanjing, Jiangsu, PR China; b Jiangsu Forestry Bureau, Nanjing, Jiangsu, PR China

**Keywords:** Ilex × Koehneana ‘Wirt L. Winn’, chloroplast genome, phylogenetic analysis

## Abstract

Ilex × Koehneana ‘Wirt L. Winn’, an important ornamental tree, has been widely distributed in southeastern China. In this study, we assembled and characterized the complete chloroplast (cp) genome of I. Koehneana to investigate its phylogenetic relationship. The whole cp genome of I. Koehneana is 157,538 bp, which contained a large single-copy (LSC) region of 87,055 bp and a small single-copy (SSC) region of 18,429 bp, and a pair of inverted repeats (IR) of 52,054 bp. A total of 137 genes, including 90 protein-coding genes, eight rRNAs, and 39 tRNAs, were identified. Phylogenetic analysis based on 74 conserved protein-coding genes revealed that I. Koehneana is closely related to I. ‘tall boy’.

*Ilex* L., in the monogeneric family Aquifoliaceae, is a woody dioecious genus cultivated as ornamentals, pharmaceutical plants, and industrial materials (Su et al. 2020). *Ilex* × *Koehneana*, also known as Koehne Holly, is an artificial hybrid between *I. aquifolium* L. and *I.latifolia* Thunb. Of them, *I. Koehneana* ‘Wirt L. Winn’ is an evergreen tree with broadleaf, and has been widely distributed in southeastern China for its ornamental, economical, and ecological values. However, due to the similar leave and flower with other species and cultivars, it is difficult to be identified and classified by morphology (Yao et al. 2016). The chloroplast (cp) genome has been extensively applied to genetic and evolutionary studies (Freitas et al. 2018). Hitherto, the cp genome of *I. Koehneana* remains undocumented. In this study, we reported and characterized its complete cp genome sequence based on Illumina sequencing data and bioinformatics analysis, which will provide more informatics data for the germplasm identification and phylogeny analysis of *Ilex* genus.

The fresh leaves of *I. Koehneana* were sampled from Nanjing Botanical Garden, Mem. Sun Yat-sen (118°49′55″E, 32°3′32″N), Nanjing, China. The voucher specimen (No. NBGJIB-Ilex-0019) was preserved at the Institute of Botany, Jiangsu Province, and Chinese Academy of Science. Genomic DNA was extracted using the GMS16011.2.1 Kit (Genmed Scientifics Inc., Wilmington, DE) according to the instructions. DNA quality and integrity were assessed using a NanoDrop spectrophotometer (Thermo Scientific; Waltham, MA) and agarose gel electrophoresis. Then, qualified DNA was used for library construction and sequencing, which was conducted using Nova-PE150 strategy with an insert-size of 350 bp at Novogene Company. As a result, 6768.01 Mb of raw data (4512.01 Mb clean data) were obtained. *De novo* cp genome assembly and annotation were conducted by NOVOPlasty version 3.3 (Dierckxsens et al. 2017) and GeSeq (Tillich et al. 2017), respectively. The annotated cp genome was deposited in GenBank database (accession no. MT435528).

The complete cp genome of *I. Koehneana* was 157,538 bp with 37.64% GC content, including a large single-copy (LSC) region of 87,055 bp, a small single-copy (SSC) region of 18,429 bp and a pair of inverted repeats (IRs, including IRa and IRb) of 52,054 bp. A total of 137 genes predicted, which contained 90 protein-coding genes, eight rRNAs, and 39 tRNAs. Among them, 15 splitting genes contained introns and two of them (*ycf3* and *clpP*) had two introns.

To investigate the phylogenetic relationship of *I. Koehneana*, phylogenetic analysis was conducted based on other 15 *Ilex* complete cp genomes and one taxa from *Couroupita guianensis* was served as outgroup (Cascales et al. 2017; Park et al. 2019; Su et al. 2020; Yao et al. 2016). Phylogenetic tree was performed based on 74 conserved protein-coding genes with those of 17 plant cp genomes by maximum likelihood method using PhyML version 3.0 software (http://www.atgc-montpellier.fr/phyml/) (Liu et al. 2019). The bootstrap values were calculated using 1000 replicates. As shown in Figure 1, the phylogenetic tree showed that *I. Koehneana* cluster into *Ilex* section and has relatively close relationship with *I.* ‘tall boy’ in this phylogenetic pattern (Figure 1). The cp genome sequence of *I. Koehneana* will provide valuable information for further analysis on population genomic and phylogenomic studies of Aquifoliaceae.

**Figure 1.**
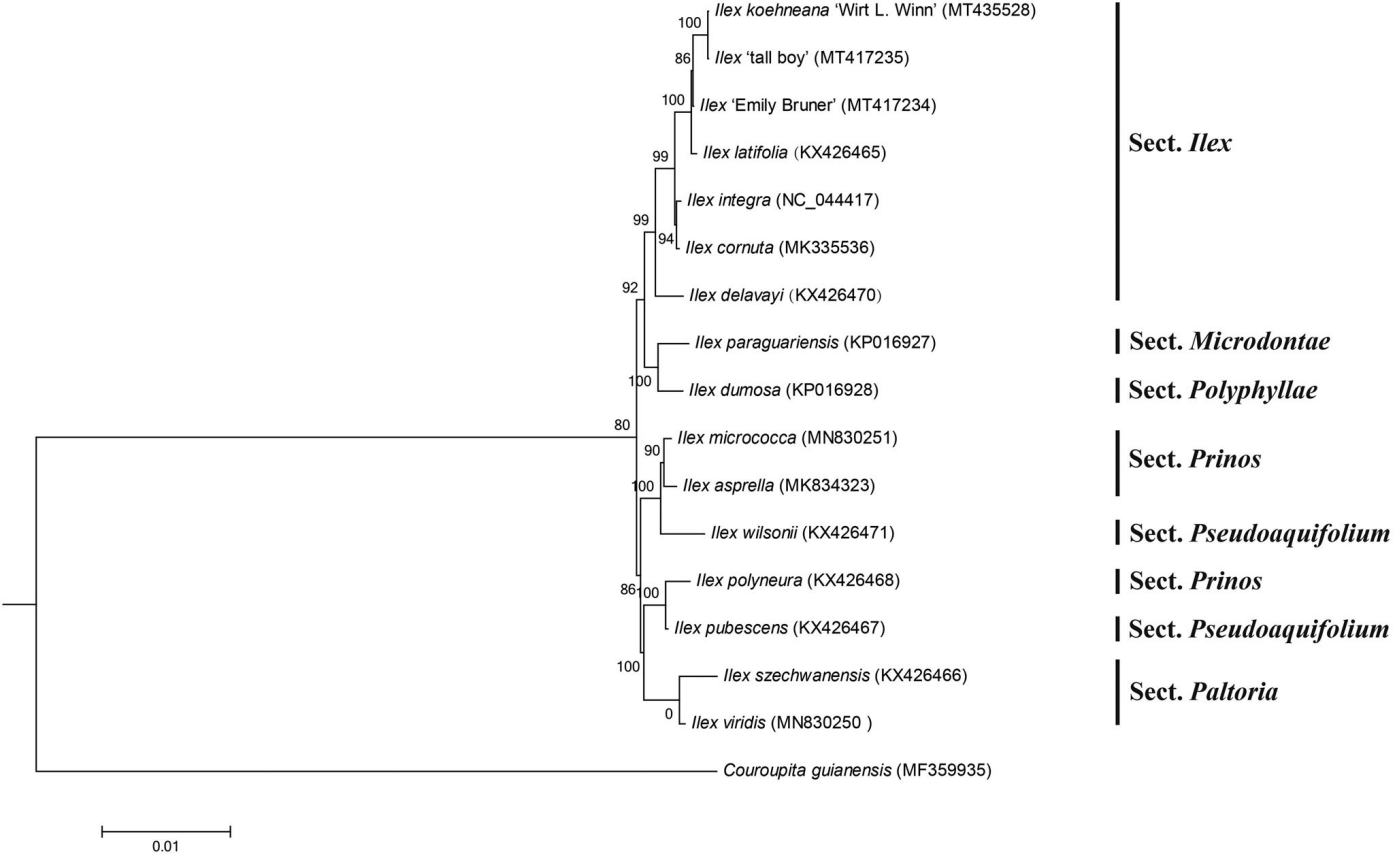
Maximum-likelihood phylogenetic tree based on the sequences of I. Koehneana and other 16 species. Section names were displayed in the right side of phylogenetic tree (Gottlieb et al. 2005; Su et al. 2020). Numbers on the nodes indicate bootstrap values.

## Data Availability

The genome sequence data that support the findings of this study are openly available in GenBank of NCBI at (https://www.ncbi.nlm.nih.gov/) under the accession no. MT435528. The associated BioProject numbers are SRR13245415.
